# Infrastructures of systems biology that facilitate functional genomic study in rice

**DOI:** 10.1186/s12284-019-0276-z

**Published:** 2019-03-14

**Authors:** Woo-Jong Hong, Yu-Jin Kim, Anil Kumar Nalini Chandran, Ki-Hong Jung

**Affiliations:** Graduate School of Biotechnology & Crop Biotech Institute, Kyung Hee University, Yongin, 17104 Korea

**Keywords:** Bioinformatics, Database, Rice, Systems biology, Functional genomics

## Abstract

Rice (*Oryza sativa* L.) is both a major staple food for the worldwide population and a model crop plant for studying the mode of action of agronomically valuable traits, providing information that can be applied to other crop plants. Due to the development of high-throughput technologies such as next generation sequencing and mass spectrometry, a huge mass of multi-omics data in rice has been accumulated. Through the integration of those data, systems biology in rice is becoming more advanced.

To facilitate such systemic approaches, we have summarized current resources, such as databases and tools, for systems biology in rice. In this review, we categorize the resources using six omics levels: genomics, transcriptomics, proteomics, metabolomics, integrated omics, and functional genomics. We provide the names, websites, references, working states, and number of citations for each individual database or tool and discuss future prospects for the integrated understanding of rice gene functions.

## Background

Systems biology is a research field that analyzes large amounts of omics data bioinformatically, constructs models for biological systems, and confirms model-driven hypotheses using biological experiments (Kitano 2002; Sauer et al. 2007). This approach provides a general biological view that is difficult to build using a single approach (Fang and Casadevall 2011). It is also a field of multi-disciplinary research that cannot be distinguished from the definition of bioinformation (Vincent and Charette 2015).

Systems biology expedites understanding of human cancer, diabetes, and Parkinson’s disease (Du and Elemento 2015; Bakar et al. 2015; Michel et al. 2016), reconstructs the metabolism pathways of microbes and algae to make cell factories (De Bhowmick et al. 2015; Nielsen and Keasling 2016), and explores synthetic biology (Andrianantoandro et al. 2006; Barrett et al. 2006; Cameron et al. 2014). In plant research, high-throughput technologies have been introduced (Yin and Struik, 2010; Glinski and Weckwerth 2006; Egan et al. 2012) and facilitate a large amount of research (Yuan et al. 2008; Fernie 2012). For example, plant systems biology has produced new understandings of metabolism (Schauer and Fernie 2006; Last et al. 2007; Sweetlove et al. 2014), stress responses (Cramer et al. 2011; Jung et al. 2013; Nakabayashi and Saito 2015), and integrative omics research (Rajasundaram and Selbig 2016). Also, together with CRISPR/Cas9 genome editing technology, plant synthetic biology has been established (Liu and Stewart Jr 2015; Baltes and Voytas 2015).

The world demand for staple crops is expected to increase by 60% from 2010 to 2050 (Fischer et al. 2014). Rice, wheat, and maize are the big three global cereals that together account for ~ 87% of all grain production worldwide. Rice is a model crop plant; it was the first plant whose whole genome information was sequenced among cereal crops (Goff et al. 2002; International Rice Genome Sequencing Project 2005), and extensive genetic studies and technological platforms have been established for functional genomic research in rice. Major goals of rice research are to identify the functional diversity of every gene and improve the crop’s agronomic traits (Zhang 2007; Zhang et al. 2008). To that end, multi-omics data have been developed using new technologies, including next generation sequencing (NGS), and many gene-indexed mutants mediated by T-DNA or transposable element insertion have been constructed (Wei et al. 2013). These resources facilitate functional genomics; as of 2017, around 3000 genes in rice had been functionally identified (Jiang et al. 2012; Yao et al. 2018). Along with ever-increasing information about wheat and maize, advancing systematic approaches in rice will help to improve the agronomic traits of other crop plants. For instance, NGS based genome-wide association studies (GWAS) have improved the resolution of quantitative trait loci (QTL) mapping in progenies of biparental crosses (Han and Huang 2013; Wang et al. 2016), systemic breeding is being based on modeling (Hammer et al. 2006; Lavarenne et al. 2018), and synthetic biology is being used for crop improvement (de Lange et al. 2018).

The data underlying systems biology are growing explosively (Stephens et al. 2015). To manage those big data efficiently, around 4800 databases have been generated (Wren et al. 2017). Many systems biology resources and well-reviewed research in rice are available (Chandran and Jung 2014; Garg and Jaiswal 2016; Li et al. 2018). However, given the proliferation, development, and updates of databases (Ősz et al. 2017; Imker 2018), an up-to-date review of the research infrastructure is essential. In this review, we report the development of tools and databases and classify them according to their major contributions to systems biology in rice. We also discuss the use of the resources and directions for further breeding and applications.

## Review

### Genomics databases and tools

Through the development of sequencing technology (Church 2006; Von Bubnoff 2008), a huge amount of rice genome data, including more than 3000 completed rice genome sequencing data, have been accumulated. Specifically, since the successful completion of the 3000 rice genomes project, research about the biological diversity of the *Oryza* genus has become available (Li et al. 2014). Many resources have been developed to provide and interpret those large genomic datasets (Table 1).

**Table 1:** List of databases for rice genomics

Category	Database	Resource link	Reference	Status	Citation/since
Genome browser	Rice Genome Annotation Project	http://rice.plantbiology.msu.edu/	10.1186/1939-8433-6-4	○^a^	850/2007
	The Rice Annotation Project	https://rapdb.dna.affrc.go.jp/index.html	10.1093/nar/gkj094	○	237/2006
	RiceGE	http://signal.salk.edu/cgi-bin/RiceGE	N/A^b^	○	N/A/2004
	RIGW	http://rice.hzau.edu.cn/rice/	10.1016/j.molp.2017.10.003	○	1/2017
	RPAN	http://cgm.sjtu.edu.cn/3kricedb/	10.1093/nar/gkw958	○	17/2016
	BGI-RIS	http://rise2.genomics.org.cn/page/rice/index.jsp	10.1093/nar/gkh085	N/A	119/2004
	OryGenesDB	http://orygenesdb.cirad.fr/index.html	10.1093/nar/gkj012	○	78/2006
	IC4	http://ic4r.org/	10.1093/nar/gkv1141	○	20/2015
GWAS	RiceVarMap v2	http://ricevarmap.ncpgr.cn/v2	10.1093/nar/gku894	○	51/2014
	SNP-Seek	http://snp-seek.irri.org/	10.1093/nar/gkw1135	○	18/2016
	Oryzagenome	http://viewer.shigen.info/oryzagenome2detail/index.xhtml	10.1093/pcp/pcv171	○	107/2006
	Ricebase	https://www.ricebase.org/	10.1093/database/baw107	○	9/2016
	HapRice	http://qtaro.abr.affrc.go.jp/qsnp/snp	10.1093/pcp/pct188	○	27/2014
	Rice imputation server	http://rice-impute.biotech.cornell.edu/	10.1038/s41467-018-05538-1	○	1/2018
	iPat	http://zzlab.net/iPat/	10.1093/bioinformatics/bty015	○	0/2018
	The GWAS viewer	http://rs-bt-mccouch4.biotech.cornell.edu/GWAS_Viewer/plot/	10.1038/ncomms10532	○	98/2016
Comparative Genomics	Gramene	http://www.gramene.org/	10.1093/nar/gkx1111	○	234/2002
	Phytozome	https://phytozome.jgi.doe.gov/pz/portal.html	10.1093/nar/gkr944	○	1749/2011
	Ensemble_rice	http://plants.ensembl.org/Oryza_sativa/Info/Index	10.1093/nar/gkx1011	○	41/2016
	PLAZA	https://bioinformatics.psb.ugent.be/plaza/versions/plaza_v4_monocots/	10.1093/nar/gkx1002	○	238/2009
	PlantGDB	http://www.plantgdb.org/	10.1093/nar/gkm1041	○	174/2004

^a^indicates that relating data is currently working

^b^indicates that currently not available

Since the early Rice Genome Annotation Project (RGAP) (Ouyang et al. 2006) and Rice Annotation Project Database (Ohyanagi et al. 2006; Sakai et al. 2013), more intensive genome browsers have been developed. The OryGenesDB (Droc et al. 2006) and rice functional genomics express database (RiceGE) both offer genome browsing using flanking sequence tag (FST) information, which provides invaluable genetic material for studying functional genomics in rice. However, only the RiceGE database was updated recently, and it provides the most up-to-date mutant information. Contrary to the aforementioned databases, which provide information about the Nipponbare genome, *japonica* subgroup, the rice pan-genome browser (RPAN) (Sun et al. 2016) and Rice Information Gateway (RIGW) (Song et al. 2018) provide genome information for various cultivars. The RPAN database deals with a pan-genome derived from the 3000 rice genomes project. It also provides variations for genes of interest among those sequences. The RIGW focuses mainly on the genome of the *indica* subgroups Zhenshan 97 and Minghui 63 because a high-quality *indica* subgroup reference genome would otherwise be absent. In addition, the Information Commons for Rice (IC4) database provides genome browser with a variety of rice genome annotations including their own annotation IC4R2.0 (IC4R Project Consortium 2015). Since IC4 is a rice knowledgebase that integrates omics data from community-contributed modules, it provides unique information such as sequence variation and transcriptome profiles, compared to other databases.

As modified forms of genome browsers, resources have been constructed to search for single nucleotide polymorphisms (SNPs) and simple sequence repeats (SSRs) found during GWAS. HapRice (Yonemaru et al. 2014), an SNP haplotype database reported in 2014, provides visualization of the allele frequency of around 3300 SNPs in a genome browser. Ricebase (Edwards et al. 2016) is a breeding and genetics platform that provides integrated genomic information about molecular markers such as SSRs. It displays the locations of SNPs, QTL, and SSRs in the Nipponbare genome generated by RGAP. Similarly, researchers can find SNP information at OryzaGenome v2 (Ohyanagi et al. 2015), RiceVarMap (Zhao et al. 2015), and the SNP-Seek database (Alexandrov et al. 2014). They respectively offer GWAS information about 446 wild *Oryza rufipogon* accessions and about 3000 rice varieties against the Nipponbare reference genome generated by the International Rice Genome Sequencing Project (Os-Nipponbare-Reference-IRGSP-1.0) (Kawahara et al. 2013). Recently, accumulated GWAS data were converted into a high-density rice array (HDRA) that covers 39,045 unique non-transposable elements in rice gene models (McCouch et al. 2016). The GWAS viewer provides a Manhattan plot of the HDRA data.

This kind of GWAS information is usually analyzed by bioinformaticians who have programming skills for command-line interfaces. Recently, however, resources have been developed to provide a graphic user interface (GUI) for GWAS study. The Intelligent Prediction and Association Tool, which was written in the Java program language, provides a GUI environment for GWAS (Chen and Zhang 2018). It can be used for rice research and is very helpful for users without programming skills. Also, the rice imputation server (Wang et al. 2018) is a web-based tool for performing genotype imputation using HDRA data.

Comparative genomics is an important approach for understanding the evolutional and functional features of genes of interest (Caicedo and Purugganan 2005; Windsor and Mitchell-Olds 2006). Using reference rice genome sequences, some databases provide information for comparative genomics analyses. Gramene is a database that specializes in comparative grass genomics (Ware et al. 2002). It is up-to-date and provides 57 reference plant genomes with other types of information. A database for comparative genomics about green plants, PlantGDB, provides expressed sequence tags for more than 16 plants (Duvick et al. 2007), but it has not been updated since 2015. In addition to PlantGDB, the PLAZA (Van Bel et al. 2017) and phytozome (Goodstein et al. 2011) databases contain 41 and 93 annotated genomes of green plant lineages, respectively. Currently, PLAZA v4.0 and phytozome v12.1.6 are available. Lastly, the Ensembl database provides the genomes of plants (including rice), bacteria, protists, fungi, and metazoa (Kersey et al. 2017).

### Transcriptomic databases and tools

Along with microarrays, improvements in transcript-assembly algorithms have led to the accumulation of transcriptome data (Martin and Wang 2011). In rice research, a variety of transcriptomic resources provide useful information (Table 2).

**Table 2:** List of databases for rice transcriptomics

Category	Database	Resource link	Reference	Status	Citation/since
Expression profile	EXPath 2.0	http://expath.itps.ncku.edu.tw/	10.1186/1471-2164-16-S2-S6	○ ^a^	11/2015
	PLEXdb	http://www.plexdb.org/index.php	10.1093/nar/gkr938	○	231/2012
	Rice eFP browser	http://bar.utoronto.ca/efprice/cgi-bin/efpWeb.cgi	10.1111/j.1365-313X.2012.05055.x	○	61/2012
	RiceXPro	http://ricexpro.dna.affrc.go.jp/	10.1093/nar/gks1125	○	166/2011
	RED	http://expression.ic4r.org/	10.1016/j.jgg.2017.05.003	○	6/2017
	TENOR	https://tenor.dna.affrc.go.jp/	10.1093/pcp/pcv179	○	31/2016
	OryzaExpress	http://plantomics.mind.meiji.ac.jp/OryzaExpress/	10.1093/pcp/pcq195	○	81/2011
	UniVIO	http://univio.psc.riken.jp/	10.1093/pcp/pct003	○	24/2013
	Genevestigator	https://genevestigator.com/gv/doc/intro_plant.jsp	10.1155/2008/420747	○	2476/2004
	Expression Atlas	https://www.ebi.ac.uk/gxa/experiments?organism=Oryza+sativa+Japonica+Group	10.1093/nar/gkv1045	○	192/2009
	CREP	http://crep.ncpgr.cn/crep-cgi/home.pl	10.1111/j.1365-313X.2009.04100.x	○	240/2010
Non coding RNA	PlantRNA	http://plantrna.ibmp.cnrs.fr/plantrna/	10.1093/nar/gks935	○	33/2012
	CSRDB	http://sundarlab.ucdavis.edu/smrnas/	10.1093/nar/gkl991	○	101/2007
	PNRD	http://structuralbiology.cau.edu.cn/PNRD/index.php	10.1093/nar/gku1162	○	72/2014
	IsomiR Bank	https://mcg.ustc.edu.cn/bsc/isomir/	10.1093/bioinformatics/btw070	○	15/2016
	miRBase	http://www.mirbase.org/	10.1093/nar/gkt1181	○	4106/2006
	PceRBase	http://bis.zju.edu.cn/pcernadb/index.jsp	10.1093/nar/gkw916	○	15/2017
	PlantcircBase	http://ibi.zju.edu.cn/plantcircbase/	10.1016/j.molp.2017.03.003	○	24/2017
	Plant rDNA database	http://www.plantrdnadatabase.com/	10.1007/s00412-012-0368-7	○	47/2012
	lncrnadb	http://www.lncrnadb.org/	10.1093/nar/gkq1138	○	452/2010
	GreeNC	http://greenc.sciencedesigners.com/wiki/Main_Page	10.1093/nar/gkv1215	○	29/2015
Co-expression analysis	PLANEX	http://planex.plantbioinformatics.org/	10.1186/1471-2229-13-83	○	25/2013
	ATTED-II	http://atted.jp/	10.1093/pcp/pcx191	○	371/2007
	PODC	http://plantomics.mind.meiji.ac.jp/podc/	10.1093/pcp/pcu188	○	32/2015
	COP DB	http://webs2.kazusa.or.jp/kagiana/cop0911/	10.1093/bioinformatics/btq121	○	66/2010
	PlantArrayNet	http://bioinfo.mju.ac.kr/arraynet/	10.1104/pp.109.139030	○	82/2009
	RiceFREND	http://ricefrend.dna.affrc.go.jp/publication.html	10.1093/nar/gks1122	○	64/2013
	NetMiner	https://github.com/czllab/NetMiner	10.1371/journal.pone.0192613	○	2/2018
Promoter analysis	PlantPAN_V2	http://plantpan2.itps.ncku.edu.tw/index.html	10.1093/nar/gkv1035	○	263/2008
	PPDB	http://ppdb.agr.gifu-u.ac.jp/ppdb/cgi-bin/index.cgi	10.1093/nar/gkt1027	○	47/2007
	PlantCARE	http://bioinformatics.psb.ugent.be/webtools/plantcare/html/	10.1093/nar/30.1.325	○	1801/2002
	PLACE	https://sogo.dna.affrc.go.jp/cgi-bin/sogo.cgi?lang=en&pj=640&action=page&page=newplace	10.1093/nar/27.1.297	○	2818/2007

^a^indicates that relating data is currently working

Typically, transcriptome databases provide genome-wide expression profiles. As a sub-part of the PlantExpress database (Kudo et al. 2017), OryzaExpress (Hamada et al. 2010) offers gene expression data obtained from 1206 samples of 34 experimental series of GPL6864 (Agilent 4X44K microarray platform) and 2678 samples of 153 experimental series of GPL2025 (Affymetrix Rice Genome Array platform). Similarly, the Collections of Rice Expression Profiling database (CREP) and Rice Oligonucleotide Array Database (ROAD) contain 190 Affymetrix GeneChip Rice Genome Arrays from 39 tissues in a single dataset and 1867 publicly available rice microarray data, respectively (Wang et al. 2010; Cao et al. 2012). The ROAD provides several tools for functional analysis, but it is under maintenance now. RiceXpro is a microarray-based database that offers three categorized data types: field/development, plant hormone, and cell−/tissue-type (Sato et al. 2012a, 2012b). Specifically, field/development expression shows the diurnal and circadian pattern of rice. In addition to expression profiles about hormones in the RiceXpro database, the uniformed viewer for integrated omics (UniVIO) database provides an intensive analysis of 43 hormone-related compounds, including a combined heatmap of the hormone-metabolome with transcriptome data (Kudo et al. 2013). To investigate genes responsive to biotic stress that are linked to the metabolism pathway, the plant expression database (PlexDB) (Dash et al. 2011) and EXPath database (Chien et al. 2015) are good resources. PlexDB provides expression data for nine pathogens from 14 plants based on pathogen GeneChip arrays, and EXPath provides tissue/organ-specific expression and gene ontology (GO) enrichment analyses coupled with the Kyoto Encyclopedia of Genes and Genomes (KEGG) pathway in six model crops, including rice. To provide more instinctive transcriptome data, the Rice eFP browser (Winter et al. 2007) uses a distinctive platform to display the expression profiles of various plants. Based on an illustration of rice tissues, it indicates expression values using color gradations.

Because RNA sequencing technology has some advantages over previously used microarray technology (Ozsolak and Milos 2011), RNA sequencing-based resources have recently been developed. The Transcriptome Encyclopedia of Rice database provides large-scale mRNA sequencing data generated with rice in a variety of conditions (Kawahara et al. 2015). This resource also provides a genome browser with transcriptome data and a search function for responsive genes. Similarly, the Rice Expression Database offers a collection of 284 high-quality RNA sequencing data and lists of housekeeping or tissue-specific genes based on that information (Xia et al. 2017). The Expression Atlas is a good platform for seeing expression profiles from recent studies (Papatheodorou et al. 2017). This resource manually curates, re-analyzes, and visualizes publicly accessible transcriptome data using a standard pipeline. Together with the Expression Atlas, the Genevestigator provides curated transcriptome data, including microarray and RNA sequencing data (Hruz et al. 2008). This client-server software also provides GUI tools for clustering genes and validating hypotheses.

The large number of expression files that have accumulated now enables co-expression analyses by clustering genes that show similar expression patterns in various situations (Aoki et al. 2007; Usadel et al. 2009a, 2009b. Several resources provide co-expression analyses based on transcriptome data. PlantArrayNet (Lee et al. 2009), the plant co-expression database (Yim et al. 2013), and the CoP database (Ogata et al. 2010) presents co-expression analyses based on large sets of microarray data, using correlation coefficients and the Confeito algorithm, which was designed to detect highly interconnected modules. These resources can provide information on the genes co-expressed with a gene of interest. Based on the RiceXPro dataset (derived from microarray series), the Rice Functionally Related Gene Expression Network Database provides two search options for co-expression analyses: single or multiple guide gene searches to identify functionally related genes in various pathways (Usadel et al. 2009a, 2009b). The recently updated ATTED-II database offers 16 co-expression platforms along with microarray and RNA sequencing-based data sources (Obayashi et al. 2017). Applying the mutual rank index as co-expression, this database provides more accurate co-expression data than earlier tools. In addition to web-based resources, standalone, genome-wide co-expression analysis tools, such as NetMiner, have been released (Yu et al. 2018). This ensemble pipeline for building a gene co-expression network can be applied to researchers’ custom RNA sequencing data.

Paying attention to similar expression patterns leads to the identification of promoter regions that participate in the regulation of gene expression (Ohler and Niemann 2001). Therefore, promoter analysis resources have been introduced. The plant cis-acting regulatory DNA elements (PLACE) (Higo et al. 1999), plant cis-acting regulatory element (Lescot et al. 2002), plant promoter database (PPDB) (Yamamoto and Obokata 2007), and plant promoter analysis navigator (Chow et al. 2015) are representative databases that provide motif information about plant cis-acting regulatory DNA elements. Among those resources, PLACE and PPDB have been updated regularly, though PPDB stopped its update service in September 2018. The Osiris database (Morris et al. 2008) was a rice-specific promoter analysis database that stored promoter sequences and predicted transcription factor binding sites for 24,209 rice genes. But it’s not available now. Alternatively, the MEME Suite (Bailey et al. 2009), a web-based motif identification tool, can help rice promoter discovery. Using these resources will enable researchers to further dissect recently reported tissue-preferred and condition-dependent rice promoters and identify key cis-acting regulatory elements (Jeong and Jung 2015).

Since it was reported that gene expression could be regulated by non-coding RNA (ncRNA), such as microRNA (miRNA) (He and Hannon 2004; Jones-Rhoades et al. 2006), the need for resources about non-coding RNA has increased. To address that demand, several databases have been developed. The Cereal Small RNA Database (Johnson et al. 2006) consists of many rice and maize small-RNA sequences obtained from pyrosequencing. The plant non-coding RNA database (PNRD) (Yi et al. 2014) and miRBase (Kozomara et al. 2018) are specialized data sources for miRNA. PNRD is plant-focused database that provides information about miRNAs, intronic long ncRNAs (lncRNA), and unknown ncRNAs from 166 plant species, including rice, and miRBase is a searchable database of reported miRNAs from 271 organisms. The recently updated version of the database provides rice a miRNA information file named with osa.gff3. The Long Noncoding RNA database (lncrnadb) provides comprehensive annotations of 287 eukaryotic lncRNAs, while the Wiki-database of plant lncRNAs (GreeNC) provides annotations of more than 120,000 lncRNAs from 37 plant species and six algae (Paytuví Gallart et al. 2015; Quek et al. 2014).

The IsomiR bank database offers multiple miRNA variants, called “isoforms of miRNAs (isomiRs)” (Zhang et al. 2016). It contains 308,919 isomiRs from eight species, including rice. Similarly, to address miRNA-related RNA, such as competing endogenous RNA (ceRNA) and circular RNA (circRNA), the plant ceRNA database (PceRBase) (Yuan et al. 2016) and plant circular RNA database (PlantcircBase) (Chu et al. 2017) have been developed. PceRBase contains potential ceRNA targets from 26 plant species, including rice, and PlantcircBase deals with 40,311 circRNA that have been predicted to be important in the transcriptional regulation of rice.

Other types of ncRNAs are also supported. The PlantRNA (Cognat et al. 2012) and plant ribosomal DNA databases (Garcia et al. 2012) support specific ncRNA information about transfer RNA and rRNA, respectively.

### Proteomics databases and tools

Transient or permanent protein–protein interactions (PPIs) are key events in cellular functions. Permanent PPIs are irreversible, whereas, transient protein complexes rapidly change their homo- or heterooligomeric states (Perkins et al. 2010). Given that approximately 80% of cellular proteins function within a complex, knowledge about PPIs provides insights into various crop traits. From the perspective of systems biology, PPIs highlight the functional pathways in large complex networks (Rao et al. 2014). To decipher the potential PPIs in rice, several resources have been constructed and host interactome datasets. These resources are broadly distinguishable in terms of the source of the embedded interactome, number of interactions, and available organisms. The interolog approach, which assumes that orthologs of interacting proteins in one organism tend to conserve their interactions in other organisms, is one method for predicting PPIs. Additional methods, such as text-mining, neighborhood analysis, co-expression analysis, fusion analysis, and co-occurrence analysis, have also been adopted to extend the interactome (Szklarczyk et al. 2017).

The Rice Interactions Viewer (RIV) database summarizes 37,112 interactions among 4567 proteins, which were deduced using the interolog approach and experimental evidence. Among those interactions, 1671 are self-interactions, and 35,441 are hetero-interactions (Ho et al. 2012). Experimental verification of the predicted interactome is supported using a dataset from the IntAct database. Though it has been a useful source, RIV is not subjected to frequent updating. To maximize interactome coverage, the STRING database includes indirect and predicted interactions and uses the widest breadth of sources available, from text-mining to computational predictions (Szklarczyk et al. 2017). STRING supports proteome information for 2031 organisms, and STRING version 10.5 hosts network connections for 26,428 *Oryza sativa japonica* subgroup proteins and 18,789 *indica* subgroup proteins. Interactions are given confidence scores that reflect biologically meaningful reproducible associations using seven evidence channels. The latest version of STRING contains Cytoscape app integration, programmatic access to the protein network, statistical analyses of the network, and user-provided interactome analyses. The predicted rice interactome network (PRIN) database annotates 76,585 non-redundant rice protein interaction pairs among 5049 rice proteins, primarily based on the interolog approach (Gu et al. 2011). Meaningful interactions within the network for the queried genes are identified by GO annotation, protein subcellular localization, and gene expression data. However, PRIN has not been updated since 2011. The database of interacting proteins in *Oryza sativa* (DIPOS) hosts 14,614,067 pairwise interactions among 27,746 proteins. DIPOS derives its PPIs from the interolog approach and domain-based predictions. However, since the database was first announced in 2011, no updates have been made to DIPOS. The IntAct Molecular Interaction Database service enables interactome data analyses derived from literature curations or direct user submissions, and it is regularly updated (Orchard et al. 2014). In addition to providing PPI information, the RiceNet database (RiceNet v2, http://www.inetbio.org/ricenet) offers two options for network prioritization including network direct neighborhood and context-associated hubs, which guide researchers to formulate their own hypotheses for future studies (Lee et al. 2015).

In addition to PPI databases, resources that feature up-to-date annotated proteomes are also essential for proteome-wide analysis. The UniProt database hosts and updates protein sequences and their annotations at regular intervals. Its current statistics indicate that the annotations of 48,916 *japonica* subgroup proteins are available in UniProt (Bateman et al. 2015). The Manually Curated Database of Rice Proteins (MCDRP) is an effort to digitize protein-related experiments. The concept of digitization addresses the demerits of text-based curation. MCDRP currently documents the details of more than 4000 experiments on more than 1800 rice proteins, and it is periodically updated (Gour et al. 2014). The OryzaPG-DB uses the proteogenomics approach to annotate the rice proteome. In proteogenomics, peptides identified in a mass spectrometry–based short-gun analysis are mapped to their genomic origins. The latest version of OryzaPG-DB (v1.1) contains an updated database design to accommodate different samples or organs, analyses such as a phosphoproteome analysis, and an application programming interface to enhance the data recovery process (Helmy et al. 2012). The plant protein annotation suit database (Plant-PrAS) provides the secondary structure for rice and *Arabidopsis* proteins. This effort is an attempt to derive protein functions based on structure. Various physiochemical properties, transmembrane helices, and the signal peptides of 208,333 proteins from six model organisms (including rice) are summarized in Plant-PrAS (Kurotani et al. 2015). In total, 40,087 records of Michigan State University (MSU) annotation and 35,908 records from the rice annotation project are integrated in Plant-PrAS.

Ortholog proteins are a valuable source of functional clues about unannotated proteins. The GreenPhyl DB v4 web utility comprises gene families manually annotated with ortholog analyses and is periodically updated. It enables comparative analysis of species and protein domains, and metabolic pathway–related information can be retrieved. The latest version of the GreenPhyl DB (v4) contains 60,647 classified rice sequences, of which 44,786 sequences have an InterPro domain (Rouard et al. 2011). The Putative Orthologous Groups 2 Database is another platform that facilitates cross-species comparative analyses for the proteomes of four species, including rice. The functionalities include graphical representation of domains, predicted protein localization, and imported gene descriptions (Tomcal et al. 2013). By receiving the proteome information from a pair of organisms, the InParanoid database estimates the ortholog groups based on the InParanoid algorithm. A standalone version of InParanoid (version 4.1) is available, and it maintains a balance between false positive and false negative entries (Sonnhammer and Östlund 2015). Similarly, the Orthologous Matrix is an interface to retrieve well-annotated ortholog groups between species and is frequently updated with new genomes (Altenhoff et al. 2018). The Panther tool allows users to classify protein sequences based on a backend library of phylogenetic trees of protein-coding genes. The library of trees is used to predict the orthologs. The coding SNP scoring tool predicts whether an amino acid substitution will affect the protein function (Mi et al. 2017). In addition, Panther provides a utility for analyzing the genes lists from high-throughput experiments. The Plant Orthology Browser is a web-based orthology analysis and annotation visualization tool that currently supports 20 genomes. The syntenic blocks are identified for a given pair of genomes using strand orientation and physical mapping (Tulpan and Leger 2017). Integrating up-to-date proteome information with the knowledge of protein subfamilies and PPIs facilitates the functional identification of novel proteins (Table 3).

**Table 3:** List of databases for rice proteomics

Category	Database	Resource link	Reference	Status	Citation/since
Proteome	MCDRP	http://www.genomeindia.org/biocuration/whatsnewver6.php	10.1093/nar/gkt1072	○ ^a^	20/2014
	OryzaPG-DB	https://github.com/MoHelmy/oryza-PG/	10.1186/1471-2229-11-63	Data available	50/2011
	Plant-PrAS	http://plant-pras.riken.jp/	10.1093/pcp/pcu176	○	10/2015
Protein Protein interaction	Rice Interactions Viewer	http://bar.utoronto.ca/interactions/cgi-bin/rice_interactions_viewer.cgi	10.1186/1939-8433-5-15	○	28/2012
	DIPOS	http://comp-sysbio.org/dipos/?id=1	10.1039/c1mb05120b	○	22/2011
	RiceNet	https://www.inetbio.org/ricenet/	10.1093/nar/gkv253	○	132/2011
	PRIN	http://bis.zju.edu.cn/prin/	10.1186/1471-2105-12-161	○	92/2011
	STRING	https://string-db.org/	10.1093/nar/gkw937	○	638/2000
Ortholog analysis	POGsDB	http://pogs.uoregon.edu/#/	10.1371/journal.pone.0082569	○	33/2007
	GreenPhyl v4	http://www.greenphyl.org/cgi-bin/index.cgi	10.1093/nar/gkq811	○	97/2006
	InParanoid	http://inparanoid.sbc.su.se/cgi-bin/index.cgi	10.1093/nar/gku1203	○	712/2005
	Plant Ortholog Browser	https://nrcmonsrv01.nrc.ca/pob/	10.1186/s12864-015-1496-2	○	4/2015
	OMA browser	https://omabrowser.org/oma/home/	10.1093/bioinformatics/btm295	○	125/2007

^a^indicates that relating data is currently working

### Metabolomics databases and tools

As sessile organisms that grow rapidly and are frequently exposed to a wide range of stress conditions, plants produce numerous metabolic compounds. One area of metabolomics profiles the small compounds that accumulate inside a cell or tissue as a result of development or stress acclimatization (Haug et al. 2013). Genetic engineering and the improvement of metabolites require knowledge about the genome-wide metabolic network. However, metabolic reconstruction, which essentially involves identifying enzymes coded by genes from the genome, mapping those genes to pathways, and then curating those pathways, will be needed (Schläpfer et al. 2017). Databases that deal with rice metabolomics and pathways facilitate functional genomic studies (Table 4).

**Table 4:** List of databases for rice metabolomics

Category	Database	Resource link	Reference	Status	Citation/since
Metabolite	MetaboLights	https://www.ebi.ac.uk/metabolights/	10.1093/nar/gks1004	○ ^a^	47/2016
	KEGG_rice	https://www.genome.jp/kegg-bin/show_organism?menu_type=pathway_maps&org=dosa	10.1093/nar/27.1.29	○	11,709/1999
	Plant Reactome	http://plantreactome.gramene.org/index.php?lang=en	10.1093/nar/gkw932	○	21/2017
	RICECYC	http://pathway.gramene.org/gramene/ricecyc.shtml	10.1093/nar/gkj154	○	52/2103
	OryzaCYC	https://plantcyc.org/databases/oryzacyc/6.0	10.1104/pp.16.01942	○	29/2017
Pathway analysis	MapMan	https://mapman.gabipd.org/home	10.1111/j.1365-313X.2004.02016.x	○	2139/2004
	Panther	http://www.pantherdb.org/	10.1093/nar/gkg115	○	526/2003
	Plant GSEA	http://structuralbiology.cau.edu.cn/PlantGSEA/index.php	10.1093/nar/gkt281	○	111/2013
	AgriGO	http://bioinfo.cau.edu.cn/agriGO/	10.1093/nar/gkq310	○	1579/2010
	KEGG Mapper	https://www.genome.jp/kegg/mapper.html	10.1093/nar/gkr988	○	3122/2017

^a^indicates that relating data is currently working

The web platform MetaboLights initiated a community data-sharing service and provides a single data access point for various metabolomic studies. Apart from its main service, the resource hosts curated metabolite information. Curated and raw experimental data from various metabolomic studies can be accessed and used to perform cross-species and cross-technique analyses. The current version includes 714 assays from 15 studies and spans more than 8 species (Haug et al. 2013). The plant metabolic network (PMN) database summarizes metabolic data from 22 species for cross-species comparative analyses and identifies 11,969 metabolic gene clusters from 18 species (Schläpfer et al. 2017). The pipeline for metabolic reconstruction consists of an enzyme annotation algorithm, pathway prediction algorithm, and semi-automated validation software. The rice metabolic database of PMN, OryzaCyc (V 6.0), currently hosts 569 pathways that consist of 3345 reactions and 2614 compounds for 6325 enzymes. Similarly, RiceCyc is a catalogue of rice biochemical pathways that have been curated and maintained by the Gramene database. Pathways in RiceCyc are based on release 5 of the TIGR-assembly (Jaiswal 2006). The pathway portal of the Gramene database, a plant reactome database, provides metabolic network, transport, genetic, signaling, and developmental pathways for 63 plant species and features rice as a reference model (Naithani et al. 2017). In addition to bulk downloading datasets from RiceCyc, datasets from the plant reactome database can also be inferred based on homology from the human reactome. Using extensive collaboration with major popular genomic and proteomic platforms, the plant reactome database hosts 222 pathways and 1025 reactions for 1173 gene products (Naithani et al. 2017). KEGG is a large hub for analyzing diverse datatypes, including metabolomics. Four databases (pathways, genes, compounds, and enzymes) perform the functionalities. The KEGG pathways database consists of manually drawn reference pathways and organism-based pathways and currently contains 530 pathway maps. Metabolites and other small-molecule information can be retrieved from the KEGG compounds database. The latest updated version includes information for 18,456 compounds (Kanehisa et al. 2017).

NGS technology has paved the way for the identification of new metabolites and has broadened knowledge about plant metabolite biosynthesis (Kim and Buell 2015). Pathway enrichment analyses of candidates from various studies is a key step in large-scale omics data analysis (Jia and Zhao 2012). A few resources are available for pathway mapping or enrichment analyses in rice functional studies. The Mapman tool helps map large omics datasets onto diagrams of metabolic pathways and various subprocesses. The tool consists of a scavenger module, the ImageAnnotator module, and the PageMan module. The scavenger module generates non-redundant ontologies and organizes transcripts, proteins, enzymes, and metabolites into functional classes. The ImageAnnotator module uses that information to organize profiling data. Users are also given the option to visualize their own experiments and customize the diagrams and maps (Usadel et al. 2009a, 2009b). Another mapping tool available with the KEGG database, KEGG mapper, functions by mapping set of genes, proteins, or small molecules onto network databases such as KEGG pathways and KEGG modules (Kanehisa et al. 2017). The two mapping options are search pathway and color pathway, which searches a list of input entities in KEGG pathways and colors those entities. GO is one of the most popular annotation methods for annotating genes from large datasets (Yi et al. 2013). To provide an enrichment analysis of agriculture species, the agriGO platform was developed (Tian et al. 2017). The current version, agriGO v2.0, supports 394 species and 865 data types and contains more visualization features and enhanced computational efficiency than previous versions. However, on the GO platform, it is difficult to increase the GO annotations and corresponding terms in consistently accumulating datasets (Tian et al. 2017). To address the low coverage of GO-annotated genes, the gene set enrichment analysis (GSEA) method was suggested. GSEA reveals the biological meaning of input genes by calculating the overlap between input genes from high throughput studies and a previously defined backend gene set. A GSEA-based web-server, PlantGSEA, uses 20,290 defined gene sets from diverse resources and enables GSEA for four model species, including rice. Using a locus id or Affymetrix probe id as input, PlantGSEA outputs an enrichment analysis with statistical support and advanced visualization. The Panther tool also provides a utility for GO analysis (Mi et al. 2017). These mapping tools provide biologically meaningful interpretation for candidates from high throughput experiments, using minimal input and making use of well-defined reference functional terms.

### Epigenomics and its resources

Recent evidence indicate that sequence variation in agronomically important genes is insufficient to address the full spectrum of plant phenotypic effects (Gallusci et al. 2017). Epigenetics also contribute to phenotypic variation and evolution by altering chromatin accessibility for DNA. Therefore, epigenomics, which includes DNA methylation and histone modification, has emerged as a new source for broadening phenotypic diversity. The high quality of genomic information in rice serves as a model for epigenetic studies in crop species. It has facilitated the extensive mapping of genome-wide methylation patterns in rice (Chen and Zhou 2013). Few web resources have been developed to access global chromatin states, segments, and genes within segments (Table 5).

**Table 5:** List of databases for rice epigenomics

Database	Resource link	Reference	Status	Citation/since
Plant chromatin state database (PCSD)	http://systemsbiology.cau.edu.cn/chromstates/	10.1093/nar/gkx919	○ ^a^	6/2018
MethBank 3.0	http://bigd.big.ac.cn/methbank	10.1093/nar/gku920	○	19/2014
RiceVarMap v 2.0	http://ricevarmap.ncpgr.cn/v2/	10.1093/nar/gku894	○	55/2014
HistoneDB 2.0	https://www.ncbi.nlm.nih.gov/research/HistoneDB2.0/	10.1093/database/baw014	○	21/2011

^a^indicates that relating data is currently working

Plant chromatin state database (PCSD) summarizes Hidden Markov Model derived chromatin states based on public and in-house epigenomic data for diverse epigenetic modifications (

 et al. 2018). In rice, PCSD provides information on 831,235 segments of 38 chromatin states from 100 datasets. The resource architecture includes search and analysis tools, and genome browser visualization and self-organization mapping results. Another interactive methylation visualization resource, MethBank, hosts information on 172 single-base resolution methylomes, 46,674 differentially methylated promoters, and 528,463 methylated CpG islands in rice (Li et al. 2018). In addition, the RiceVarMap database incorporates chromatin accessibility data generated by Assay for Transposase-Accessible Chromatin using sequencing (ATAC-Seq) or DNase-seq (Zhao et al. 2015) data while the HistoneDB database presents manually curated sets of histone sequences, their classifications and their variance in animal and plant species including rice (Draizen et al. 2016).

### Phenomics databases and tools

To address the bottleneck in plant functional genomics, the efforts in identifying phenotypes associated with genotypes have been made to systematically collect phenotype data by recording agronomic trait data through sophisticated non-invasive imaging, spectroscopy, robotics, high-performance computing facilities, and phenomics databases (Mir et al. 2019). Phenomics databases and tools aim to record data on agronomic traits, such as plant development, architecture, photosynthesis, growth, or biomass productivity (Table 6). Databases and tools such as TRY (http://www.try-db.org) (Kattge et al. 2011) and Plant Image Analysis (https://www.plant-image-analysis.org/) (Lobet et al. 2013) include rice phenome information related to root and grain traits. In addition, phenotyping tools such as SmartGrain, General Image Analysis of Roots (GiA Roots) and Panicle TRAit Phenotyping (P-TRAP) are specialized for seed shape, root system architecture and panicle structure phenotyping, respectively (Faroq et al. 2013; Galkovskyi et al. 2012; Tanabata et al. 2012). The phenomics data generated have been used to identify genes or QTL and to provide association mapping data or GWAS for crop improvement. High-throughput phenotyping platforms facilitate the screening of large germplasms or mapping populations, and phenome-wide associated study (PheWAS) approaches would facilitate GWAS, which reveals previously reported genotype-phenotype associations and identifies novel ones.

**Table 6:** List of databases for rice phenomics

Database	Resource link	Reference	Status	Citation/since
Plant image analysis	https://www.plant-image-analysis.org/	10.1186/1746-4811-9-38	○ ^a^	122/2013
SmartGrain	Stand alone program	10.1104/pp.112.205120	○	132/2012
GiA Roots	http://giaroots.biology.gatech.edu/	10.1186/1471-2229-12-116	○	82/2012
P-TRAP	http://bioinfo.mpl.ird.fr/index.php?option=com_content&view=article&id=102&Itemid=2	10.1186/1471-2229-13-122	○	15/2013
TRY	http://www.try-db.org	10.1111/j.1365-2486.2011.02451.x	○	1275/2011

^a^indicates that relating data is currently working

### Integrated omics and specialized gene family databases

Apart from the databases and tools for the individual omics levels described above, specialized resources have also been constructed for specific gene families or to provide summaries of previously characterized rice genes (Table 7). The overview of functionally characterized genes in rice online (OGRO) database summarizes all the genes that have been functionally characterized and published in various articles using the manual check approach. As of 31 March 2012, the functions of 702 rice genes were elucidated and included in the OGRO database, with information about 11 additional genes provided in the gene information table (Yamamoto et al. 2012). However, OGRO is no longer being updated. To overcome the disadvantage of expert curation in extracting rice functional information from the literature, RiceWiki, a type of community platform, was developed. RiceWiki is an open-content community platform curation resource that hosts rice information and contains more than 1000 manually curated genes (Zhang et al. 2014). In addition, gene expression profiles and scientific articles are also integrated into the resource. The funRiceGenes database is designed to function similar to the OGRO database and contains information about 2800 functionally characterized genes and 5000 members from different families of rice. These genes constitute 19.2% of the predicted coding genes (Yao et al. 2018). The information is retrieved based on text-based extraction and manual curation. This resource also includes a gene network of 214 interaction networks for 1310 genes.

**Table 7:** List of databases that integrate omics or specialized gene familes

Category	Database	Resource link	Publication	Status	Citation/since
Integrative omics	CARMO	http://bioinfo.sibs.ac.cn/carmo/	10.1111/tpj.12894	○^a^	15/2015
Characterized genes	OGRO	http://qtaro.abr.affrc.go.jp/ogro/table	10.1186/1939-8433-5-26	○	75/2012
	funRiceGenes	https://funricegenes.github.io/	10.1093/gigascience/gix119	○	3/2017
	RiceWiki	http://wiki.ic4r.org/index.php/Main_Page	10.1093/nar/gkt926	○	19/2014
Abiotic stress	DroughtDB	http://pgsb.helmholtz-muenchen.de/droughtdb/	10.1093/database/bav046	○	25/2015
	STIFDB	http://caps.ncbs.res.in/stifdb2/	10.1093/pcp/pcs185	○	80/2013
	RiceSRTFDB	http://www.nipgr.res.in/RiceSRTFDB.html	10.1093/database/bat027	○	38/2013
Domain specific	Grassius	https://grassius.org/index.php	10.1104/pp.108.128579	○	180/2009
	RKD 2	http://ricephylogenomics-khu.org/kinase/	10.1186/s12284-016-0106-5	○	6/2016
	RGTD	http://ricephylogenomics.ucdavis.edu/cellwalls/gt/	10.1093/mp/ssn052	○	75/2008
	RGHD	http://ricephylogenomics.ucdavis.edu/cellwalls/gh/	10.3389/fpls.2013.00330	○	14/2013
	PlnTFDB	http://plntfdb.bio.uni-potsdam.de/v3.0/	10.1093/nar/gkp805	○	412/2009
	RiTE	https://www.genome.arizona.edu/modules/xnews/article.php?storyid=254	10.1186/s12864-015-1762-3	○	23/2015
Localization	MPIC	http://webapps.plantenergy.uwa.edu.au/applications/mpic/	10.1093/pcp/pcu186	○	19/2015
*Indica* specific	RICD	http://server.ncgr.ac.cn/ricd/	10.1186/1471-2229-8-118	○	29/2008

^a^indicates that relating data is currently working

Because transcription factors (TFs) are central to the regulation of gene expression and so the most actively studied of all gene families, multiple resources have been developed to address them. The Stress Responsive Transcription Factor Database (STIFDB V2) is a hub that integrates abiotic and biotic stress-responsive TF genes in rice and *Arabidopsis* (Naika et al. 2013). In addition to stress-related genes and annotation data, the current version of STIFDB includes 38,798 stress signals and TF binding sites predicted using the stress-responsive transcription factor (STIF) algorithm. Similarly, in addition to *cis*-regulatory elements information, RiceSRTFDB provides TF expression under drought and salinity stress conditions at various developmental stages (Priya and Jain 2013). The phylogenomic approach is a method of integrating multi-omics data into the phylogenetic context to identify the functionally redundant or dominant genes in rice (Jung et al. 2015). The updated rice kinase database (Version 2) enables a phylogenomic analysis of rice kinase genes (Chandran et al. 2016). Similar databases are available for rice glycosyltransferase and glycoside hydrolase families (Cao et al. 2008; Sharma et al. 2013).

### Functional genomics databases and tools

Rice functional genomic research is aimed at deciphering the genes that control agronomic traits. An effective way to identify gene function is to analyze phenotypic differences in mutants compared with wild-type plants using forward and reverse genetics. Since the International Rice Functional Genomics Consortium proposed the goal of discovering every gene’s function by 2020 (Zhang et al. 2008), more than 200,000 mutants with FSTs have been collected and summarized (Krishnan et al. 2009; Chang et al. 2012; Jung et al. 2008; Wang et al. 2013; Chandran and Jung 2014). Genome-wide mutant libraries generated through the insertion of transfer DNA (T-DNA), transposons, or Tos17 contain at least one insertion for around 60% of nuclear genes and 68% of genic regions, covering more than half of the rice genome (Hong and Jung 2018). PFG-FST in Korea provides the largest number of indexed mutants in rice: 106,100 FSTs mapped to an RGAP v6 annotation (Jung and An 2013). Another T-DNA insertional mutant pool employing an enhancer trap system covered 85,315 FSTs (Zhang et al. 2006). Fast-IC4R Project Consortium 2015 used to generate a mutant library in the Kitaake rice variety (Li et al. 2016; Li et al. 2017a, 2017b, 2017c). Advances in high-throughput sequencing have made it possible to characterize 1504 mutants at the whole-genome level, which has identified 91,513 mutations affecting 32,307 genes. This advanced technical tool will enable the indexing of all available mutants, including chemically and physically generated populations, in a cost-effective way. Another unique database, RiceFOX, has been developed by the ectopic expression of full-length rice cDNA into *Arabidopsis* (Sakurai et al. 2011). More than 30,000 independent transgenic *Arabidopsis* lines have been screened under various conditions, providing a gain-of-function systematic characterization tool.

The gene-indexed genome-wide mutant pool serves as a powerful tool in functional gene characterization, making both forward and reverse genetics easy and facilitating systems biology. The functional genomics databases, such as RiceGE and OryGenesDB, integrate mutant resources to visualize mutant information from various sources within the genome browser (Droc et al. 2006). Recently, RiceGE updated 12 mutant resources, including datasets produced in KitBase (Table 8) based on MSU version 7 in 2018, which has greatly promoted rice functional studies. Intelligent platforms such as funRiceGenes (Yao et al. 2018), OGRO (Yamamoto et al. 2012), and RiceWiki (Zhang et al. 2014) continue to provide functional characterized genes and their publications with related traits, providing timely information. Currently, a total of 3148 genes have been functionally characterized, and around 5000 members of different gene families (https://funricegenes.github.io/) account for about 20% of all predicted rice genes. Functional studies of cloned rice genes have revealed the genes that determine yield, grain quality, resistance to biotic and abiotic stresses, nutrient-use efficiency, and successful reproductive development, which all have potential utility in crop improvement (Jiang et al. 2012; Bai et al. 2018; Li et al. 2018; Yao et al. 2018). The identification of gene function in OGRO (http://qtaro.abr.affrc.go.jp/) uses the overexpression and knockdown approach (about 50%), mutants (about 40%), and natural variations (about 7%). A comprehensive mutant and genomic database of allelic variations in the natural population will explore the effects of functional genes on traits. A user-friendly, integrated tool containing all of the diverse mutant resources and phenomics data on a single platform will enhance the value of indexed mutant libraries for functional research.

**Table 8:** List of rice gene indexed mutant databases

Database	Types	Number of FSTs	Resource link	Reference	RiceGE	Orygene	Citation/since
RMD	T-DNA	85,315^a^	http://rmd.ncpgr.cn/	10.1111/j.1365-313X.2006.03001.x	○ ^b^	○ ^b^	174/2007
POSTECH-RISD	T-DNA	107,171^a^	N/A	10.1046/j.1365-313x.2000.00767.x	○	○	689/2000
TRIM	T-DNA	59,804^a^	http://trim.sinica.edu.tw/	10.1007%2Fs11103–006-9093-z	○	○	206/2007
SHIP T-DNA	T-DNA	10,381^a^	N/A	10.1038/cr.2009.15	○	○	38/2009
CIRAD	T-DNA	29,263^a^	http://oryzatagline.cirad.fr	10.1111/j.1365-313X.2004.02145.x	○	○	137/2004
CSIRO	Ac-Ds	611^a^	N/A	10.1111/j.1467-7652.2004.00081.x	○	○	46/2004
EU-OSTID	Ac-Ds	1315^a^	http://orygenesdb.cirad.fr	10.1007/s11103-005-8532-6	○	○	71/2005
UCD	Ac-Ds	17,730^a^	N/A	10.1111/j.1365-313X.2005.02570.x	○	○	94/2005
NIAS	Tos17	77,740^a^	https://tos.nias.affrc.go.jp	10.1105/tpc.012559	○	○	466/2003
GSNU	Ac-Ds	1072^a^	N/A	10.1111/j.1365-313X.2004.02116.x	○	○	104/2004
Zhejiang Univ	T-DNA	741^a^	http://genomics/zju.edu.cn/ricetdna	10.1046/j.1365-313X.2003.01860.x	○	N/A	226/2004
KitBase	Irradiation	94,252^a^	http://kitbase.ucdavis.edu/	10.1105/tpc.17.00154	○	N/A	11/2017
N/A	CRISPR-Cas9	25,604	N/A	10.1016/j.molp.2017.06.006	N/A	N/A	23/2017
N/A	CRISPR-Cas9	91,004	N/A	10.1016/j.molp.2017.06.007	N/A	N/A	19/2017
RiceFOX	Rice cDNA	18,000	http://ricefox.psc.riken.jp/	10.1093/pcp/pcq190	N/A	N/A	60/2010

^a^Updated on 2018 Jan 18 from RiceGE. ^b^ indicates that relating data is currently working

A molecular tool, the clustered regularly interspaced short palindromic repeats-associated nuclease 9 (CRISPR/Cas9) gene editing system, has recently emerged as a powerful tool for targeted mutagenesis and functional genomics research in numerous organisms, including all major crops (Cong et al. 2013; Shan et al. 2013; Ma et al. 2016). In rice, the percentage of homozygous or bi-allelic mutants in the T0 generation is almost 90%, providing high efficiency mutations at the intended target sites (Ma et al. 2016). Two recent studies demonstrated the efficient editing of target genes in mutant libraries created via genome-scale CRISPR/Cas9 mutagenesis in rice (Meng et al. 2017; Lu et al. 2017). The studies generated almost 100,000 targeted loss-of-function rice mutants, which represent a key resource, and the technique could be adapted for other crops. Compared with traditional mutagenesis, the CRISPR/Cas9 mutant system facilitates rapid and inexpensive generation of potential causal mutations for a phenotype by identifying the gRNA sequence of the corresponding target. Suitable target design tools for corresponding vectors have been developed, such as CRISPR-GE (Xie et al. 2017), CRISPR-PLANT (Xie et al. 2014), CRISPR-P (Liu et al. 2017a, 2017b), E-CRISP (Stemmer et al. 2015), and CRISPR RGEN (Bae et al. 2014). In addition, the tools facilitate the screening for phenotypes associated with lethal alleles in the T0 generation, which was a limitation of insertional heterozygous mutant lines. In addition, advances in CRISPR technology will further improve the resources available for functional studies. For example, nuclease-dead Cas9 (dCas9) fused with a transcriptional activation domain could be used to generate gain-of-function mutants (Li et al. 2017a), and multiple target editing tools could reveal the functions of genes exhibiting functional redundancy.

## Conclusions

**Fig. 1:**
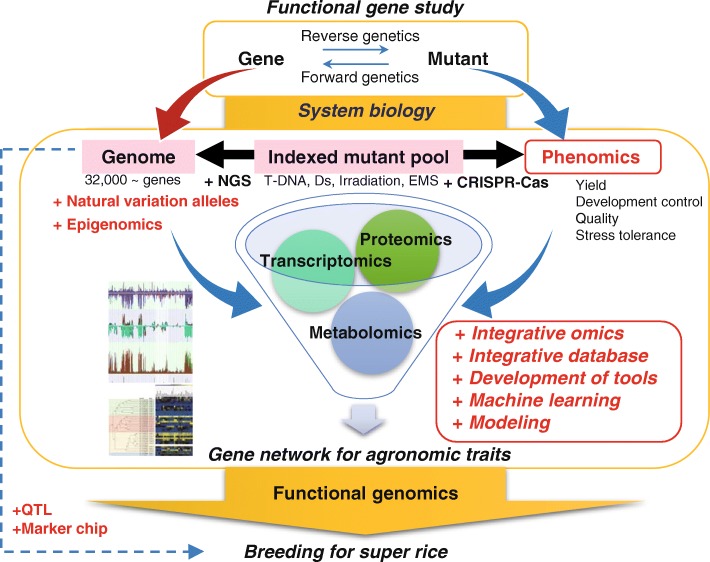
Infrastructures of systems biology to facilitate gene discovery for enhanced agronomic traits in rice

Improving rice yield largely depends on functional analyses of genes that contribute to important agronomic traits, such as grain yield and stress tolerance. Recently generated datasets will facilitate rapid gene discovery and provide the evolutionary insights needed to feed the future. The targeted gene editing and indexed mutant libraries made possible by CRISPR technology will accelerate high-throughput, forward genetic screening for desired traits and crop improvement. In spite of tremendous efforts to create a genome-wide mutant population, determining the relationship between the genotype and phenotype of a mutant remains a bottleneck for functional genomics. Genes with redundant functions in a gene family, genes that are functional only under some specific conditions such as stress or tissues, and critical genes that cause lethality are all challenges that need to be met. Therefore, systematic characterization tools that include phenomics are needed to establish a platform of mutant resources. An integrative omics platform or functional network providing access to all bioinformatics data, including tools, knowledge, and resources, will enable researchers and breeders to adopt new tools and resources for forward/reverse genetics and breeding approaches (Fig. 1).

## Abbreviations

ceRNA: Competing endogenous RNA
circRNA: Circular RNA
CREP: Collections of rice expression profiling database
CRISPR/Cas9: clustered regularly interspaced short palindromic repeats-associated nuclease 9
DIPOS: Database of interacting proteins in *Oryza sativa*
FST: Flanking sequence tag
GO: Gene ontology
GSEA: Gene set enrichment analysis
GUI: Graphic user interface
GWAS: Genome-wide association studies
HDRA: High-density rice array
isomiRs: Isoforms of miRNAs
KEGG: Kyoto Encyclopedia of Genes and Genomes
lncRNA: Long non-coding RNA
MCDRP: Manually curated database of rice proteins
miRNA: microRNA
MSU: Michigan State University
ncRNA: Non-coding RNA
NGS: Next generation sequencing
OGRO: Overview of functionally characterized genes in rice online
PceRBase: Plant ceRNA database
PheWAS: Phenome-Wide Associated Study
PLACE: Plant cis-acting regulatory DNA elements
PlantcircBase: Plant circular RNA database
Plant-PrAS: Plant protein annotation suit database
PlexDB: Plant expression database
PMN: Plant metabolic network
PNRD: Plant non-coding RNA database
PPDB: Plant promoter database
PPIs: Protein–protein interactions
PRIN: Predicted rice interactome network
QTL: Quantitative traits loci
RGAP: Rice genome annotation project
RiceGE: Rice functional genomics express database
RIGW: Rice information gateway
RIV: Rice interactions viewer
ROAD: Rice oligonucleotide array database
RPAN: Rice pan-genome browser
SNP: Single nucleotide polymorphism
SSR: Simple sequence repeats
STIF: Stress-responsive transcription factor
STIFDB: Stress responsive transcription factor database
T-DNA: Transfer DNA
TF: Transcription factor
UniVIO: Uniformed viewer for integrated omics
